# Age-dependent development of the interincisive suture: a CT-based study on prevalence and dimensions

**DOI:** 10.1007/s12565-025-00901-x

**Published:** 2025-10-18

**Authors:** Julian J Graef, Moritz Staeber, Michael J Schmeisser, Sven Schumann

**Affiliations:** 1 Institute of Anatomy, University Medical Center of the Johannes Gutenberg University Mainz, Mainz, Germany; 2 Focus Program Translational Neurosciences, University Medical Center of the Johannes Gutenberg University Mainz, Mainz, Germany; 3 Institute of Anatomy, Brandenburg Medical School, Neuruppin, Germany; 4 Faculty of Health Sciences, Joint Faculty of the Brandenburg University of Technology Cottbus-Senftenberg, the Brandenburg Medical School Theodor Fontane and the University of Potsdam, Postdam, Germany; 5 Institute of Anatomy, Brandenburg Medical School, Geschwister-Scholl-Straße 36, Haus A, 14776 Brandenburg an der Havel, Germany

**Keywords:** Sutura interincisiva, Premaxilla, Craniofacial development, Orthodontics, Hard palate

## Abstract

The interincisive suture (sutura interincisiva) connects the left and right premaxilla at the midline of the anterior palate and plays a role in craniofacial development. Despite its anatomical and developmental importance, postnatal changes in this suture remain understudied. Here, we investigated the age-dependent growth and closure of the interincisive suture using CT scans from 384 patients aged 0–94 years. Suture presence, length, and width were assessed in five-year age groups. Results show a progressive closure of the suture with age, with complete obliteration in most individuals over 35. Rates of open interincisive sutures in older patients are up to 25%. Sex differences emerged, with females showing a higher rate of suture closure in early years but a higher rate of open sutures in old age. A strong negative correlation was observed between age and both suture width (*r* = −0.545, *p* < 0.001) and length (*r* = −0.530, *p* < 0.001). The findings highlight similarities with, but also distinctions from, other midfacial sutures such as the midpalate suture and the incisive suture. Clinical implications include timing considerations for orthodontic interventions and improved understanding of midfacial growth patterns.

## Introduction

### Anatomical description

**Fig. 1:**
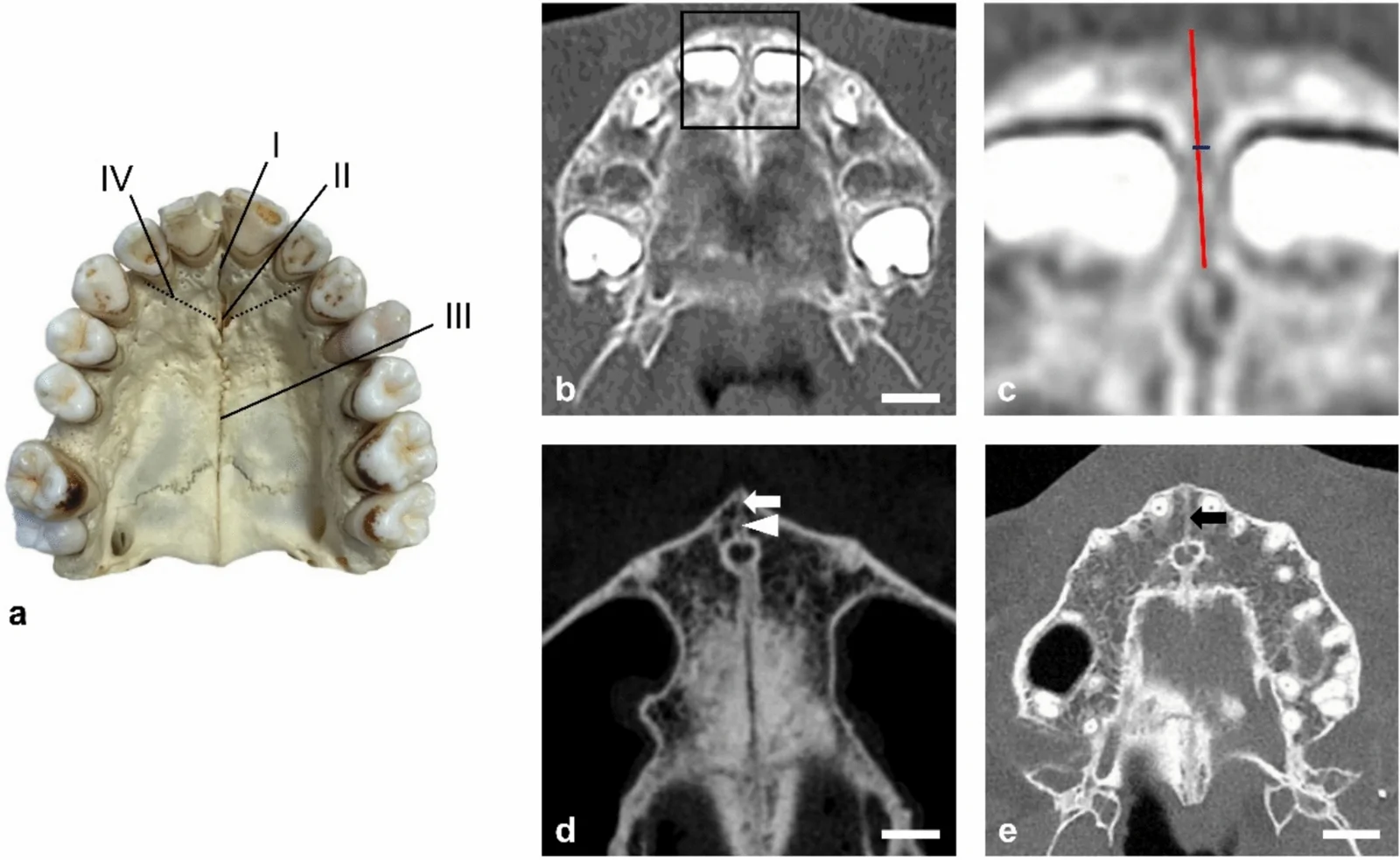
Overview of palatal sutures (**a**) and examples for an open (**b**, **c**), partially closed (**d**) and closed (**e**) interincisive suture. (**a**) Palatal sutures in a macerated dry adult human skull from the anatomical collection Mainz. I: Interincisive suture = sutura interincisiva = sutura interpremaxillaris. II: Foramen incisivum. III: Midpalate suture = sutura palatina mediana. IV: Incisive suture = premaxillary–maxillary suture = sutura incisiva (completely closed in this specimen). Premaxillary sutures might be used as umbrella term for interincisive suture and incisive suture. (**b**) CT image of a 3-year-old male with an open interincisive suture. (**c**) Higher magnification of the marked region in **b**. The red line and the blue line indicate where the length (red) and wide (blue) of the suture were measured. (**d**) CT image of a 27-year-old female with a partially closed interincisive suture. The closed part is marked with an arrow, the open part with an arrowhead. (**e**) CT image of a 75-year-old male with a closed interincisive suture (arrow). Bar in **b** and **d**: 5 mm. Bar in **e**: 10 mm

The interincisive suture (= sutura interincisiva = sutura interpremaxillaris) (Fig. 1 a) is the midline connection between the left and right premaxilla (= incisive bone = os incisivum) at the anterior palate (Inouye 1912). The interincisive suture extends from the upper central incisors to the incisive foramen and continues superiorly into the vomer, where it becomes indistinguishable from the midline suture of the vomer (Delaire 1976). In adult humans, the premaxilla is typically fully fused with the maxilla. Historically, the distinction between the premaxilla and maxilla was a subject of debate. Anatomists such as Coiter (1573) and Goethe (1784) were among the first to describe an “incisive bone” in humans (Goethe 1784; Trevizan and Consolaro 2017). Later research demonstrated the independent prenatal development of the premaxilla. While the majority of authors support premaxillary independence, some researchers continue to dispute this theory (Barteczko and Jacob 2004). Consequently, the interincisive suture has received limited attention in scientific literature, with only a few references in the 1800s and sparse publications in the 1900s (Delaire 1976; Barteczko and Jacob 2004).

### Development

As the fusion site of the two incisive bones, the interincisive suture plays a central role in the development of the anterior palate and face. Cells forming the incisive bones originate from the neural crest (Jiang et al. 2002). Facial development begins during the fourth week of embryogenesis (Jiang et al. 2006). At this stage, the paired medial nasal prominences start to form, and between the fourth and fifth week, the philtrum and upper lip begin to develop. Within the oral cavity, several paired prominences emerge and fuse to form the primary palate or intermaxillary segment (Hinrichsen and Beier 1993). The ossification of the intermaxillary segment is not fully understood yet, with descriptions of one, two, or even up to six ossification centers (Felber 1917; Barteczko and Jacob 2004).

Research focusing specifically on the postnatal interincisive suture is limited. Most authors suggest that the suture closes during early childhood and highlight its role in lateral maxillary growth (Nicol et al. 2020). Other sutures, such as the premaxillary–maxillary suture (= sutura incisiva = incisive suture), which connects the maxilla and premaxilla, have received more attention. The premaxillary–maxillary suture shows partial patency up to the age of 12, allowing for anterior–posterior expansion during adolescence (Trevizan et al. 2018).

### Clinical relevance

Although it has received limited attention in the literature, the interincisive suture plays a role in craniofacial development and might have clinical relevance. The premaxilla serves as a structural keystone of the midface, and abnormalities in its growth or its fusion with the maxilla can contribute to malocclusions and midline dental anomalies (Savoldi et al. 2018). In orthodontics, most expansion techniques focus on the midpalate suture (= sutura palatina mediana). Understanding the closure status and remaining growth potential of facial sutures is critical for treatment planning (Cerritelli et al. 2022). Early or delayed fusion of the interincisive suture may influence transverse maxillary growth and dental spacing, although current evidence supporting this is limited. Modern imaging techniques, such as computed tomography (CT) and cone beam computed tomography (CBCT) now allow in vivo evaluation of this suture, providing valuable insights for both diagnosis and surgical planning.

### Study aim and research questions

Given the limited knowledge regarding the postnatal development of the interincisive suture, this study aims to investigate and illustrate its morphological changes across the human lifespan. Specifically, we seek to determine whether the suture is open or closed across human life and to analyze its length and width over time. Based on the large dataset analyzed, we aim to provide meaningful insights and lay the foundation for future research in this underexplored area.

## Materials and methods

### Data

Retrospectively pseudonymized, Hounsfield standardized cranial CT scans from the University Medical Center Mainz were used. Ethics approval was obtained before data gathering (2024–1747). The dataset consists of patients who underwent CT scans for medical reasons. The specific clinical indication for each scan was not disclosed to the examiners. Data was reviewed for their suitability for the measurements. Patients whose entire anterior palate was not visible or intact, who showed any pathologies of the skull, or whose scans did not contain a sufficient number of images of the palate, were excluded. A total number of 384 patients was included in this study. The mean age was 42.9 for males and 49.9 females. Patients were grouped in five-year age cohorts. The number of patients in each five-year age cohorts and sex distribution is shown in Table 1.

**Table 1:** Sample characteristics across age groups and sex

Age group	Male	Female	Total
0–4	17	14	31
5–9	11	4	15
10–14	12	11	23
15–19	10	7	17
20–24	18	3	21
25–29	11	8	19
30–34	17	7	24
35–39	9	8	17
40–44	11	6	17
45–49	10	12	22
50–54	4	8	12
55–59	14	10	24
60–64	14	7	21
65–69	15	10	25
70–74	12	8	20
75–79	9	11	20
80–84	11	8	19
85–89	6	13	19
90–94	6	12	18
Total	217	167	384

Patient cohort statistics are presented with distinctions by age group and sex. Each age group included a minimum of 12 and a maximum of 31 patients. In total, 384 patients were analyzed, comprising 217 males and 167 females

### Measurement

The selected images were measured using SECTRA Workstation (Linköping, Sweden). The images were aligned on axial and sagittal planes as described by Savoldi et al. (2022). The axial plane was manually aligned parallel to the hard palate. The sagittal plane was aligned passing through the anterior nasal spine (ANS) and the posterior nasal spine (PNS) and perpendicular to the axial plane. The frontal plane was adjusted to ensure consistency in measuring interincisive suture width across patients. It was aligned at the root tips of the central incisors. For teeth that were still erupting, the middle of the apex was used. For edentulous jaws, the estimated position of the central incisors was used.

The occurrence of an open interincisive suture was analyzed jointly by two examiners. Disagreements between the two examiners were discussed, and a final decision was reached by consensus. A suture was classified as open if it appeared as a continuous hypodense structure extending fully or partially between the ANS to the incisive foramen. The hyperdense lateral borders were identified as the cortical bone of the incisive bone (Fig. 1b). A closure of the suture was defined as the lack of a continuous hypodense structure along this region, indicating suture obliteration (Fig. 1d, e).

The following measurement of an open interincisive suture was performed by one researcher. The interincisive suture length was measured along the axial plane from the most anterior visible point at the anterior nasal spine to the incisive foramen. If the suture was only partially open (Fig. 1c), the open portions were measured. The suture width was measured along the axial plane at the position of the cemento-enamel junction of the central incisors.

### Data analysis

Data were analyzed using jamovi software (The jamovi project 2025). A binary logistic regression was conducted to evaluate the effect of age on the presence of the interincisive suture. A correlation matrix between age and width and length was performed. Spearman’s correlation was used to identify a linear relationship between age and length or width.

## Results

**Fig. 2:**
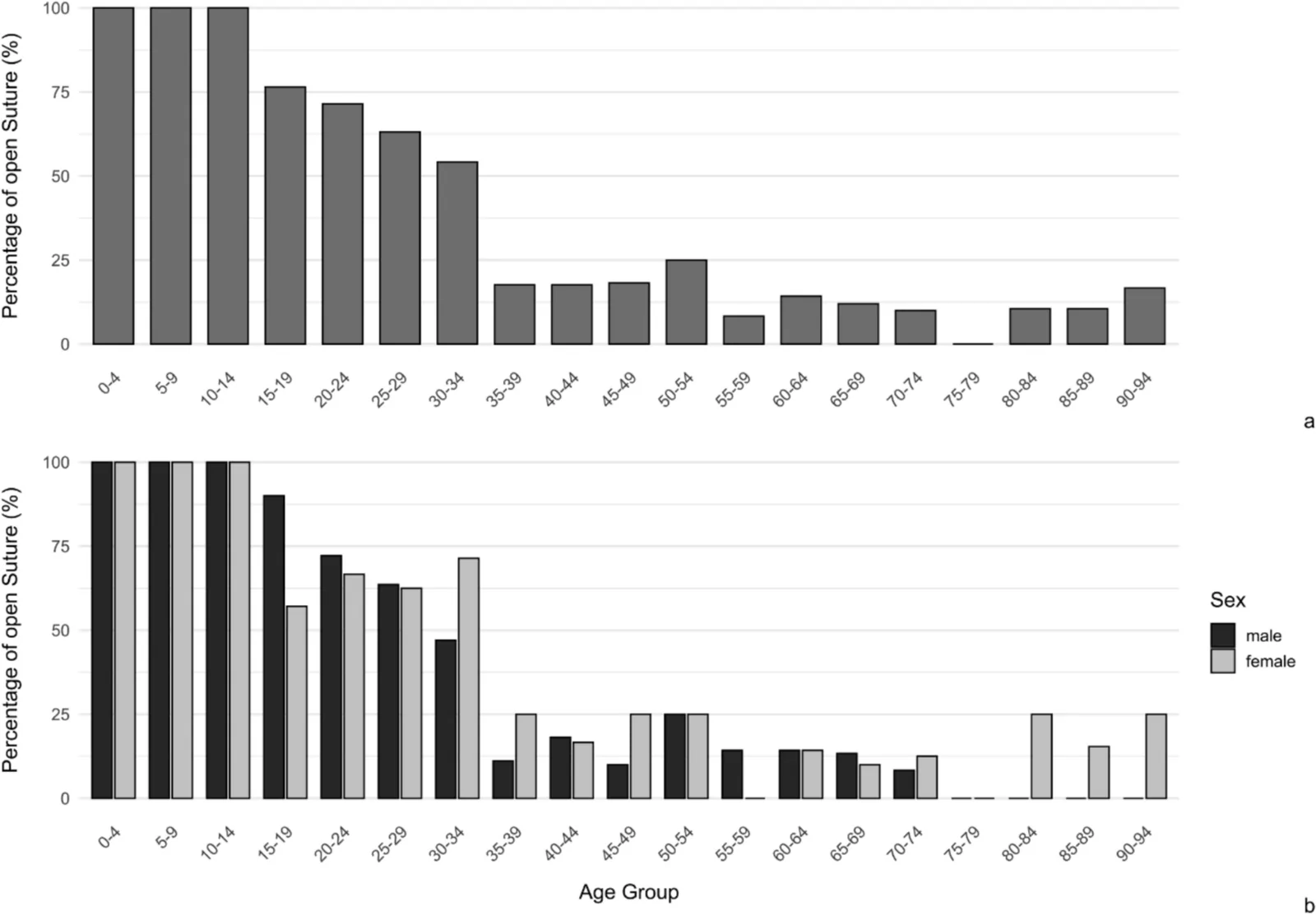
Relative distribution of open sutures (%) across different age groups (**a**) and separated by sex (**b**). (**a**) The prevalence is highest in younger age groups (0–9 years) and gradually declines with age. A slight increase in suture presence is observed in older age groups, particularly among individuals aged 90–94 years. (**b**) When separated by sex, the distributions are similar for most age groups, but notable sex differences emerge between ages 15 and 34. Whereas males show a continuous decrease in this phase, a similar trend was not visible in females. Between ages 80 and 94, open interincisive sutures were only observed in females

The interincisive suture was open in all individuals up to the age group of 10–14, after which the occurrence of open sutures decreased with age. This decrease was gradual between age groups 15–19 and 30–34, decreasing from 76 to 54%. A sharp drop followed between the age groups 30–34 and 35–39 (Fig. 2a). In older age groups, the rate of open sutures remains low, fluctuating slightly, peaking at 25%, and dropping to 0% (Fig. 2a). When testing for the correlation between age and suture occurrence, the overall model was statistically significant (*χ*^2^(3) = 206, *p* < 0.001) and explained 39.9% of the variance in suture presence (McFadden *R*^2^ = 0.399). The model also demonstrated high discriminatory ability, with an area under the receiver operating characteristic curve (AUC) of 0.891, indicating very good classification performance. Age was a significant negative predictor of an open interincisive suture (OR = 0.909, *p* < 0.001).

Comparing the rate of open sutures by sex, no differences were observed up to the 10–14 age group. A difference emerged in the 15–19 age group, where 90% of male patients had an open suture compared to 57% of female patients (Fig. 2b). The rate in male steadily declines up to the age of 35–39, with a sharp drop between the 30–34 and 35–39 age groups. In contrast, rate of open sutures remains relatively stable until age group 35–39 in female. After this period, both male and female patients exhibited fluctuations in the prevalence of open sutures, ranging between 0 and 25%. In the age groups from 75–79 until 90–94, open incisive sutures were only observed in females.

Across all ages sex was not a significant predictor (OR = 0.304, *p* = 0.054). However, the interaction between age and sex was significant (OR = 1.041, *p* = 0.007), indicating that the effect of age on suture presence differed by sex.

**Fig. 3:**
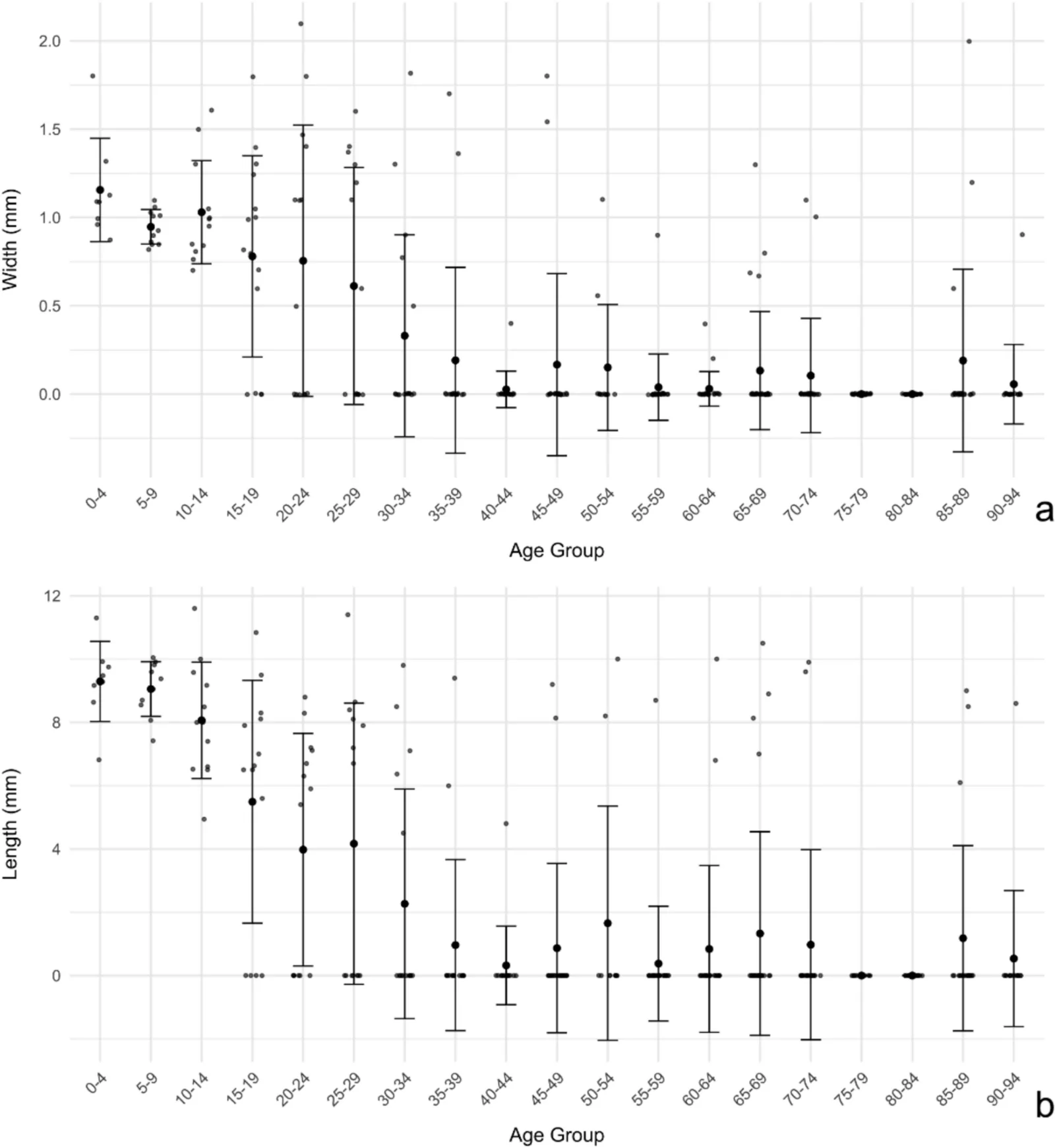
Suture width (**a**) and length (**b**) across different age groups. Mean suture width (**a**) and mean suture length (**b**) by age groups. Each dot represents an individual measurement, while the error bars indicate the mean and standard deviation for each age group. Both width and length measurements demonstrate a decreasing trend with increasing age, with greater variability observed in younger and older age groups

The mean suture width decreases with age (Fig. 3a). For ages up to 14, the mean suture width is 1 mm with a mean deviation of less than 0.25 mm. In age groups over 14 up to 39, the mean deviation increases to a range between 0.57 and 0.77 mm, while the suture width steadily decreases. Specifically, the mean width of 1 mm in the 10 to 14 age group reduces to a mean width of 0.25 mm for those above 35. The data shows a strong negative correlation between age and suture width (*r* = −0.545, *p* < 0.001). When differentiating by sex, the data reveals a strong negative correlation in males (*r* = −0.603, *p* < 0.001) and a moderate negative correlation in females (*r* = −0.495, *p* < 0.001).

The mean suture length consistently decreases with age from 15 to 35 (Fig. 3b). For ages up to 14, the mean length ranges from 8.03 to 9.29 mm. The mean deviation ranges between 0.86 and 1.84 mm. Beyond 35, the mean length decreases to values between 1.33 and 0 mm with a mean deviation up to 3.7 mm. The data shows a strong negative correlation between age and suture length (*r* = −0.530, *p* < 0.001). When analyzed by sex, the data indicates a strong negative correlation in males (*r* = −0.582, *p* < 0.001) and a moderate negative correlation in females (*r* = −0.483, *p* < 0.001).

## Discussion

### Age- and sex-dependent ossification of the interincisive suture

This study aimed to investigate the postnatal development and closure of the interincisive suture using CT scan measurements in a large sample (*n* = 384), with sample sizes in previous studies ranging from 28 to 200 patients (Korbmacher et al. 2007; Katti et al. 2020). Our findings demonstrate a clear trend toward suture closure with increasing age. In neonates and young children, the suture appeared as a clearly visible junction between the two premaxillary segments. This appearance gradually diminished in adolescents and adults, with the suture predominantly obliterated in individuals over 35 years of age. These results confirm that the interincisive suture undergoes age-dependent closure, progressing from full patency in children to closure in approximately 80% of adults.

In younger individuals with an open suture, the gap between the premaxillary segments exceeded 1 mm in width and reached lengths of up to 9.29 mm, extending from the incisive foramen to the region of the central incisors. With increasing age, this space progressively narrowed and shortened. In most adults, the suture showed no measurable length or width, indicating complete bony fusion.

Our data also suggest sex-related differences in the timing of interincisive suture closure. While sex alone was not a significant factor in the rate of suture closure, the interaction between sex and age revealed significant differences between males and females. While boys and girls displayed similar rates of open sutures during early childhood, differences became apparent during pubertal and post-pubertal years. Female patients tended to exhibit earlier suture closure compared to males in these age groups. Female rates in early adulthood stay consistent, while males seem to have a steadier decline over the years. Above the age of 35 the rates of open sutures in males and females are very similar. Higher rates of open sutures in females can be seen in high age groups above 80 years of age. Although modest, these sex differences suggest the influence of biological factors, such as hormonal regulation, that contribute to suture maturation in a sex-specific manner (Andrew et al. 2022). Sex differences in suture closure have previously been described, with females showing an earlier onset of closure (Pryor 1928; Wu et al. 2022). One possible contributing factor is estrogen, a hormone known to play a key role in bone metabolism (James et al. 2009). In younger individuals, estrogen plays a role in maintaining bone homeostasis by inhibiting bone resorption and supporting balanced remodeling. After menopause, the decline in estrogen levels leads to increased bone resorption and reduced bone formation, which may influence craniofacial bone metabolism and potentially slow suture closure in older females compared to males (Singh et al. 2010).

### Comparison with other midfacial sutures

When comparing the results to the postnatal development of other cranial sutures, in particular the midpalate suture, many similarities emerge. The midpalate suture lies posterior to the interincisive suture, contributing together to the transversal growth of the palate (Haghanifar et al. 2017). Some authors defined the midpalate suture as the suture in full length including the interincisive suture, using different categories to define the closure state (Korbmacher et al. 2007; Angelieri et al. 2013; Haghanifar et al. 2017; Katti et al. 2020; Oliveira et al. 2021). The stage of closure in these studies were labelled from A to E, with A to C meaning a visible suture on the whole maxilla including the premaxilla, D meaning partial closure, and E meaning no suture visible at all. Despite these differences in definition, we see these studies as a suitable data source to compare them to our findings.

The studies mentioned showed that closure typically starts between the ages of 11 and 20 (Angelieri et al. 2013; Haghanifar et al. 2017; Katti et al. 2020). Some authors reported sex-related differences in closure timing, with females showing closure already between the ages of 11 and 15 (Angelieri et al. 2013). The suture is generally open in early age groups and progressively closes, with complete closure commonly observed between the ages of 40 and 50. Only minimal rates of open midpalate suture have been observed in individuals over 60 years of age, although the findings were limited by sample size and age range (Angelieri et al. 2013; Haghanifar et al. 2017; Katti et al. 2020; Oliveira et al. 2021). In contrast, we observed an open interincisive sutures in older patients in around 20%. Therefore, we consider the midpalate suture and the interincisive suture are distinct sutures that may influence each other but ultimately exhibit independent pre- and postnatal development.

### Comparative anatomy

The differences in suture closure patterns may have both evolutionary and functional significance. In apes, monkeys, and many other mammals, the premaxilla persists as a separate bone, typically retaining a distinct and often prominent interincisive suture (Koyabu 2023). The premaxilla is more prominent in these species due to its dominant role in forming the snout (Higashiyama et al. 2021). In non-human primates, premaxillary sutures remain open throughout infancy and adolescence, and often remain partially patent into adulthood. For example, in chimpanzees, the anterior nasal spine is less prominent than in humans, possibly due to incomplete closure of the interincisive suture (Mooney and Siegel 1991).

Some authors propose functional explanations for these differences in premaxillary development across species (Ashley-Montagu 1935). Unlike reptiles, mammals generally lack true cranial kinesis (Arnold 1988). However, mammals with a persistent interincisive suture often exhibit increased mobility and deformation capacity in the anterior upper jaw. In rodents, the independent premaxilla supports continuous incisor growth and resists the mechanical stresses of gnawing (Krentzel 2019). Incomplete ossification of the premaxillary sutures provides the necessary flexibility to absorb these forces. Similarly, pigs display mobility of the premaxilla during mastication (Rafferty et al. 2003). Their snouts are frequently used for rooting in soil, and increased premaxillary flexibility may help them withstand the associated mechanical forces.

Fossil evidence from young Neanderthals suggests that their premaxillary sutures remained open longer than in anatomically modern humans (Maureille and Bar 1999). Neanderthals possessed a robust midface with large noses and incisive regions (Rak 1986). The prolonged patency of premaxillary sutures, including the interincisive suture, may have played a role in the development of these features.

### Methodological considerations and limitations

When assessing the reliability of the results, the methodological framework plays a critical role. This includes the selection of the patient cohort, the use of CT, and the measurement procedures. Our first age group (0–4) is characterized by the first dentition, while the subsequent age groups are primarily defined by the transition to permanent dentition and the onset of puberty (Yang and Kiyak 1998). These developmental phases are essential for a study like ours, but recruiting a sufficient number of patients from these younger age groups is a significant challenge. The use of X-ray imaging, particularly CT scans, is discouraged in children due to the heightened sensitivity to radiation during early development (Pancherza 2000). CT imaging involves relatively high radiation exposure and is typically performed only after a careful assessment of its risks and benefits (Kukolj et al. 2023). In most cases, CT scans are justified only in trauma settings where immediate diagnostic clarity is essential. As a result, access to CT data from younger patients is limited, which may lead to sampling bias and restrict the generalizability of findings to the broader population. Even in older age groups, CT scans were only performed when medically indicated, often due to suspected or known pathological conditions in the head and neck region. Although cases with visible fractures or maxillary abnormalities were excluded, the potential influence of undiagnosed or unrelated conditions on suture development cannot be fully excluded. The same applies to prior therapies or orthodontic treatments, which could not be assessed due to data anonymization.

Additional limitations stem from the technical and procedural aspects of measurement. Measurement inaccuracies may result from varying scan resolutions, slice thicknesses, and artifacts introduced by metal dental work (Matkovic et al. 1994; Hidayati and Mamile 2021). Manual measurements, although based on anatomical landmarks, are inherently subjective and susceptible to interpretation bias.

However, CT scans were crucial for this study. Modern CT scanners offer high resolutions, a high soft tissue contrast with high anatomical accuracy compared to CBCT scans (Lechuga and Weidlich 2016). MRI scans provide better visualization of soft tissue, but the lack in image resolution and the difficulty in detecting bone structures, compared to CT scans, make it challenging to accurately assess sutures. (Heye et al. 2012). Furthermore, the widespread use of CT imaging in daily clinical practice allows for a broader population from which to recruit patients.

Another limitation of this study is its cross-sectional design, which limits the ability to track individual developmental changes over time. This limitation could be addressed in future studies by using longitudinal imaging data, ideally starting from early childhood. In addition, routinely collected orthodontic records, including panoramic radiographs, may provide valuable insights into suture development during critical growth periods. The consistent timing of such records could improve comparability across age groups and enable a more dynamic understanding of suture maturation.

The decision to stratify the study population into detailed age groups and by sex was made to capture age-dependent changes in suture closure more precisely, particularly during adolescence and early adulthood. However, this approach inevitably led to small sample sizes in several subgroups, which may have contributed to large standard deviations and limited the statistical power to detect significant differences between some individual groups. As a result, statements regarding differences between specific subgroups must be interpreted with caution. Nevertheless, a general trend in suture closure was consistently observed across the dataset, and this trend was confirmed as statistically significant in the overall analysis. This trade-off between anatomical resolution and statistical robustness is acknowledged as a limitation of the study, and the interpretation of subgroup results was made within the context of this broader pattern.

### Clinical relevance

During early development, the open interincisive suture permits both transverse and longitudinal growth of the premaxilla. This has implications for the timing of interventions such as maxillary expansion (Wang et al. 2022). Closure of the suture by adolescence limits the potential for orthodontic manipulation (Trevizan and Consolaro 2017). Persistent patency may influence incisor alignment and the overall success of orthodontic treatments. In surgical procedures for widening the palate, only the midpalate suture is typically used to facilitate expansion. Awareness of the prolonged patency of the interincisive suture supports the rationale for this approach. Conversely, earlier fusion of the interincisive suture may contribute to treatment failure (Kraut 1984).

### Future research directions

Looking ahead, several promising research topics emerge. Further studies might analyze genetic and molecular factors that regulate the development of the interincisive suture and compare them to those of other midfacial sutures. Research focusing on sex differences could help to clarify the subtle differences we observed between males and females. Further research in evolutionary and comparative anatomy is also warranted. Data on midfacial development in other species remain limited, and the premaxilla as an independent bone is underrepresented in standard human anatomical descriptions. Comparative studies between humans and other animals could provide stronger evidence for the developmental and functional independence of the premaxilla and the interincisive suture.

## Conclusion

Overall, the study provides a thorough insight into the postnatal development of the interincisive suture, demonstrating its gradual obliteration with age and revealing sex-related differences in its closure pattern. Our findings suggest that the interincisive suture develops independently from other palate sutures and highlight its possible relevance for midfacial growth. This work supports a more nuanced understanding of craniofacial development and assists further anatomical, clinical, and evolutionary research.

## Data Availability

Please contact the corresponding author for data requests.
